# A Multi-Cohort and Multi-Omics Meta-Analysis Framework to Identify Network-Based Gene Signatures

**DOI:** 10.3389/fgene.2019.00159

**Published:** 2019-03-19

**Authors:** Adib Shafi, Tin Nguyen, Azam Peyvandipour, Hung Nguyen, Sorin Draghici

**Affiliations:** ^1^Department of Computer Science, Wayne State University, Detroit, MI, United States; ^2^Department of Computer Science and Engineering, University of Nevada, Reno, NV, United States; ^3^Department of Obstetrics and Gynecology, Wayne State University, Detroit, MI, United States

**Keywords:** multi-cohort, multi-omics, meta-analysis, subnetwork identification, GBM, LGG

## Abstract

Although massive amounts of condition-specific molecular profiles are being accumulated in public repositories every day, meaningful interpretation of these data remains a major challenge. In an effort to identify the biomarkers that describe the key biological phenomena for a given condition, several approaches have been developed over the past few years. However, the majority of these approaches either (i) do not consider the known intermolecular interactions, or (ii) do not integrate molecular data of multiple types (e.g., genomics, transcriptomics, proteomics, epigenomics, etc.), and thus potentially fail to capture the true biological changes responsible for complex diseases (e.g., cancer). In addition, these approaches often ignore the heterogeneity and study bias present in independent molecular cohorts. In this manuscript, we propose a novel multi-cohort and multi-omics meta-analysis framework that overcomes all three limitations mentioned above in order to identify robust molecular subnetworks that capture the key dynamic nature of a given biological condition. Our framework integrates multiple independent gene expression studies, unmatched DNA methylation studies, and protein-protein interactions to identify methylation-driven subnetworks. We demonstrate the proposed framework by constructing subnetworks related to two complex diseases: glioblastoma and low-grade gliomas. We validate the identified subnetworks by showing their ability to predict patients' clinical outcome on multiple independent validation cohorts.

## 1. Introduction

Due to the rapid advances in high-throughput technologies, massive amounts of biological data are currently available in public repositories for many diseases. These biological data include various *omics* profiles such as genomic, transcriptomic, metabolomic, and proteomic data, each of which describes different aspects of cellular mechanisms. Understanding the *mechanism of action* for a given disease from these vast resources and subsequently identifying reliable biomarkers that can predict the patients' clinical outcome has become a major challenge.

Over the last decade, the number of disease-specific biomarkers reported by different research groups has increased exponentially. However, biomarkers obtained from different studies of the same condition often show very poor agreement with each other (Ein-Dor et al., 2006). As a result, only a few of the proposed biomarkers are currently in clinical use (Burke, 2016). One of the primary reasons for this *reproducibility crisis* is that many of the conventional biomarker discovery methods simply rely on selecting a list of candidate genes based on their differential expression across the given phenotypes (disease vs. normal, treated vs. non-treated, subtype A vs. subtype B, etc). Better results can be obtained by utilizing gene interaction data that became available with the introduction of publicly available sources such as pathway knowledge databases [e.g., KEGG (Ogata et al., 1999; Kanehisa and Goto, 2000), Reactome (Matthews et al., 2009)] or protein-protein interaction databases [e.g., HPRD (Peri et al., 2003), STRING (Szklarczyk et al., 2016)].

Numerous computational methods have been proposed that aim to address the above-mentioned challenge by integrating known interactions between the genes and subsequently identifying network-based markers using different strategies. For instance, PinnacleZ (Chuang et al., 2007) and DIAMOnD (Ghiassian et al., 2015) use greedy algorithm-based techniques; jActiveModules (Ideker et al., 2002) and COSINE (Ma et al., 2011) utilize evolutionary algorithms; HotNet (Vandin et al., 2011) and ResponseNet (Lan et al., 2011) use diffusion-flow based techniques; EnrichNet (Glaab et al., 2012) employs random walk algorithms; etc. These network-based approaches have been reviewed elsewhere (Mitra et al., 2013; Nguyen T. et al., 2018). It has been demonstrated in various disease conditions [e.g., breast cancer (Chuang et al., 2007), colorectal cancer (Shi et al., 2012; Shafi et al., 2015), and ovarian cancer (Jin et al., 2015)] that network-based markers are more reproducible and reliable for predicting patients' clinical outcome than individual gene biomarkers. Although somewhat useful, the majority of these methods construct their networks using only one transcriptomic experiment. Therefore, they are unable to account for the heterogeneity that may arise due to the biological and technical variabilities present in independent studies of a given disease (Drăghici et al., 2006; MAQC Consortium, 2006).

In order to account for the data heterogeneity present in the individual studies, several meta-analysis approaches have been proposed over the past years. These can be divided into two main categories. The approaches in the first category use multiple sample-unmatched studies of the same data type (e.g., mRNA) and aim to identify robust gene signatures that can distinguish disease-affected individuals from the healthy ones. These approaches include classical *p*-value-based approaches (Fisher, 1925; Stouffer et al., 1949; Nguyen et al., 2016c), modern effect-size-based approaches (Haynes et al., 2017) and rank aggregation-based approaches (Pihur et al., 2009). However, these approaches may not be suitable for revealing the mechanism of action for a given disease since they do not account for the heterogeneity that is present across multiple data types (mRNA, miRNA, DNA methylation, etc.). The approaches in the second category combine sample-matched studies from multiple data types and provide biomarkers that can capture data heterogeneity present across the *omic* layers. Integrating such information from multiple data types is essential for obtaining a comprehensive overview of the given biological system and thought to provide better prognostic markers (Berger et al., 2013; Kristensen et al., 2014; Nguyen et al., 2016b). For instance, it has been shown that integrating miRNA and mRNA expression profiles results in greater statistical power and better understanding of the underlying disease phenomena, both in the context of biomarker discovery (Volinia and Croce, 2013; Wotschofsky et al., 2016) and pathway analysis (Calura et al., 2014; Vlachos et al., 2015; Alaimo et al., 2016; Diaz et al., 2016). More recently, it has been demonstrated that the integration of long non-coding RNA (lncRNA) and mRNA plays an important role in revealing pathogenetic mechanisms of a given condition (Lin et al., 2014; Liu et al., 2018). However, these approaches require the same group of individuals to be present for each of the experiments coming from different *omic* layers. Thus, they fail to utilize the information from dozens of independent studies containing thousands of samples for a given disease that is currently available in public repositories such as Gene Expression Omnibus (GEO) (Barrett et al., 2005), TCGA [http://cancergenome.nih.gov] or ArrayExpress (Rustici et al., 2013).

DNA methylation has been recognized to play a crucial role in cancer progression (Esteller, 2008; Parrella, 2010). An increasing number of computational approaches have been published in recent years for the identification of methylation-based biomarkers (Gevaert et al., 2015; Hao et al., 2017; Hong et al., 2017; Shafi et al., 2018). However, to the best of our knowledge, none of the current approaches is able to identify network-based gene signatures considering the data heterogeneity among the independent DNA methylation and gene expression studies. The approach presented in this manuscript bridges this gap.

Here we propose a multi-cohort and multi-omics meta-analysis framework that is able to integrate unmatched mRNA and DNA methylation data obtained from many different independent studies, and subsequently identify network-based signatures that can capture putative mechanisms of a given disease. We apply our proposed framework on nine independent datasets related to glioblastoma (GBM) containing a total of 622 samples and eight independent studies related to low-grade glioma (LGG) containing a total of 1,787 samples. The identified network-based signatures are validated based on their ability to predict the patients' clinical outcome for 1,269 samples from four completely independent validation datasets. This is done by clustering the patients included in the validation datasets using perturbation clustering (Nguyen et al., 2017b), which identifies the correct number of clusters present in the data and groups the patients accordingly. The signatures extracted from the proposed framework are then compared with 10 other previously published gene signature panels related to GBM and LGG. For both diseases, the network-based signatures identified by our proposed framework are able to separate patients associated with poor survival from other individuals with significant Cox *p*-values and outperform the other compared signatures. This suggests that the proposed framework is able to provide better prognostic biomarkers compared to the existing ones.

## 2. Materials and Methods

The goal of the proposed framework is to identify reliable network-based gene signatures by integrating independent experiments obtained from multiple data types. The framework takes three types of inputs: (i) mRNA datasets, (ii) DNA methylation datasets, and (iii) known gene interaction networks. The mRNA and DNA methylation datasets can be completely independent, which means that they can be obtained from different experiments performed in different laboratories and can include samples from different cohorts of patients. The gene interaction network is a graph in which the nodes represent genes and the edges represent interactions between them. This information can be obtained from any resources that describe the known gene-gene interactions such as KEGG, Reactome, STRING, or HPRD.

Each mRNA or methylation dataset is represented by a matrix in which the rows represent the measured genes and the columns represent the samples included in the given study. The value in each cell reflects the measured expression or methylation level of a gene for a particular sample. Each dataset includes samples from two given phenotypes such as disease vs. healthy, treated vs. non-treated, disease subtype A vs. disease subtype B, etc.

**Figure 1:**
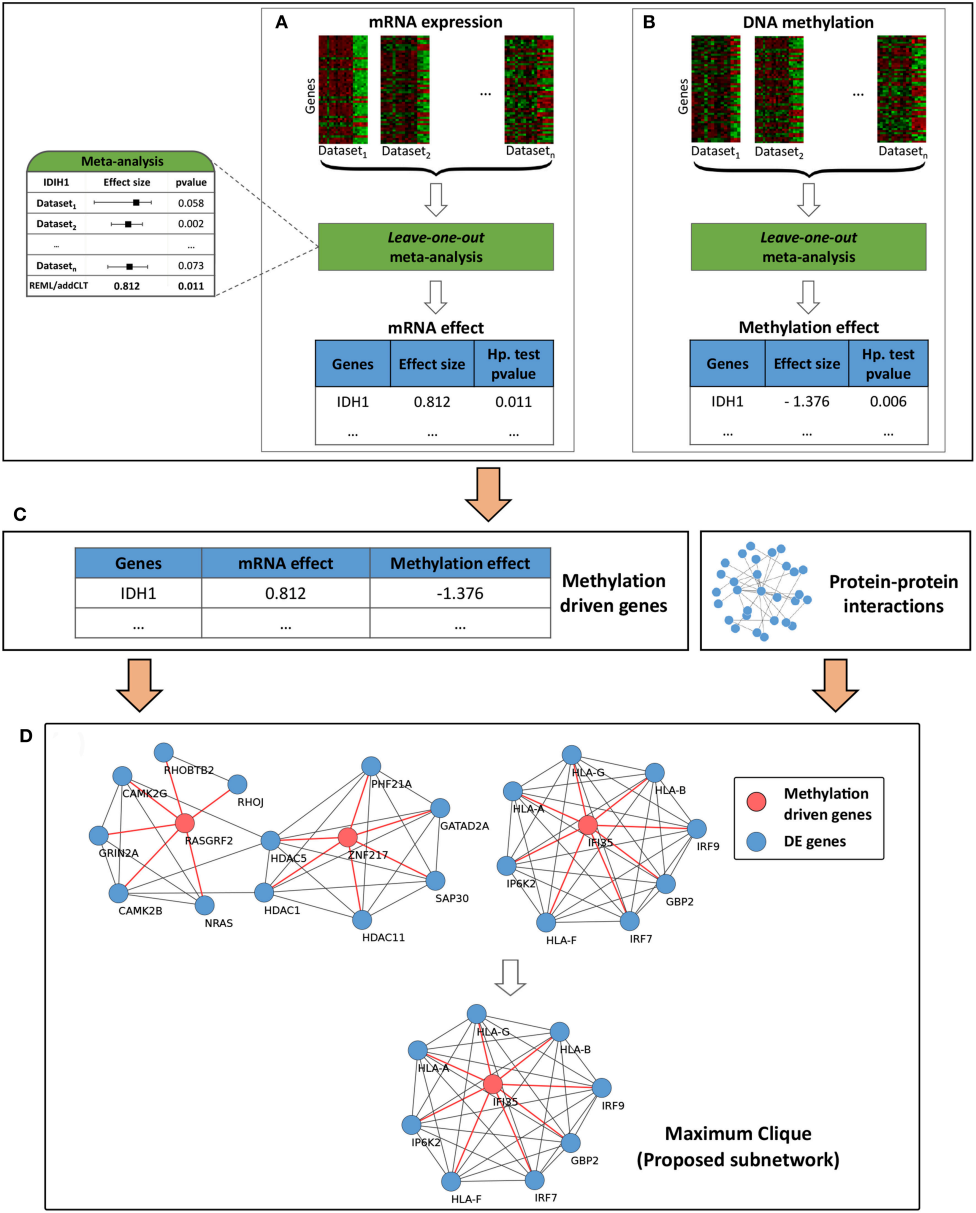
Overall workflow of the proposed framework. **Module (A)** takes multiple independent mRNA datasets and performs a leave-one-out meta-analysis to identify reliable differentially expressed genes (DEGs). Similarly, **module (B)** takes multiple independent DNA methylation datasets and identifies differentially methylated genes (DMGs). DEGs and DMGs are then systematically integrated in **module (C)** to identify methylation-driven genes (MDGs). Finally in **module (D)**, the MDGs are used as inputs in a network propagation algorithm to identify the proposed subnetworks.

The overall workflow of the proposed framework is divided into four main modules (Figure 1). The first two modules, described in section 2.1, account for the variability across the individual datasets coming from the same data type, while the third and fourth modules, described in section 2.2, account for the variability across the data types (mRNA and methylation) and integrate network information into the framework in order to identify impacted subnetworks. Briefly, the first module takes the given list of mRNA datasets as input and performs a meta-analysis to identify the genes that are differentially expressed across the given phenotypes. Due to the heterogeneity present in the individual mRNA datasets, the identified list of genes might be significantly impacted by a single study, and hence might not represent the true list of genes impacted for the given condition. Therefore, a *leave-one-out* (Friedman et al., 2001) meta-analysis is carried out to make the list of genes more reliable. The second module takes the given list of methylation datasets as input and utilizes the same meta-analysis pipeline to identify the genes that are differentially methylated across the given phenotypes. The third module combines the results obtained from the first two modules and identifies the genes that are driven by their methylation profiles. This module essentially integrates information obtained from two *omic* layers (transcriptomic and epigenomic) and takes into account the heterogeneity that may arise across these layers. Finally, the fourth module incorporates the known interactions among the genes and identifies the subnetworks that are affected by the methylation-driven genes.

### 2.1. Multi-Cohort Meta-Analysis

This section describes the first and second modules of the framework (Figures 1A,B). The meta-analysis pipeline proposed here utilizes both classical *p*-value-based and modern effect-size-based meta-analysis to calculate gene level statistics. The backbone of this algorithm is an extended version of the meta-analysis framework proposed in one of our previously published works (Nguyen et al., 2016a). The overall pipeline consists of three steps: (i) obtaining *p*-values from classical hypothesis testing, (ii) obtaining effect sizes and their *p*-values and (iii) combining the two types of *p*-values to calculate the final gene level statistics. The first two steps are independent of each other and can be performed concurrently.

At first, two-tailed *p*-values are calculated for all genes across all studies by performing a classical hypothesis testing. A moderated *t*-test provided by limma (Smyth, 2005) is utilized for this purpose. This can also be replaced with other classical tests such as two sample *t*-test, paired *t*-test, etc.

If the input matrix contains discrete values (e.g., data obtained from RNA-seq experiment or bisulfite sequencing experiment), regression-based approaches such as Poisson, quasi-Poisson or negative binomial regression models should be used instead (Robinson et al., 2010; Anders et al., 2012; Klein and Hebestreit, 2015; Shafi et al., 2018). The two-tailed *p*-values are then converted to one-tailed (left- and right-tailed) *p*-values. Gene level *p*-values generated by the individual studies are then combined by using *addCLT* (Nguyen et al., 2017a), an *additive approach* (Edgington, 1972) based on the *Central Limit Theorem* (Kallenberg, 2002) that is robust against outliers. For each gene, this *p*-value represents the chance of observing its combined differential expression (or methylation) just by chance.

To estimate the effect size, we first calculate the standardized mean difference (SMD) of each gene across all studies. Considering SMD instead of the raw mean difference is crucial since the expression (or methylation) levels within each study might be scaled differently. In this work, we use Hedge's *g* (Hedges and Olkin, 2014) as the SMD to measure expression (or methylation) changes between the two given phenotypes. Central tendencies for the effect sizes are calculated using the random-effect model and the REstricted Maximum Likelihood (REML) algorithm (Viechtbauer, 2010). Next, we calculate the z-scores and left- and right-tailed *p*-values of the z-scores to estimate the probability of observing such effect sizes just by chance. This overall estimated effect size represents the expression (or methylation) change of a gene under the effect of the given condition.

In the third step, we combine the two types of evidence (one obtained from classical hypothesis testing, another from estimating the effect sizes) using a conservative maxP (Wilkinson, 1951) method. We are using this conservative statistic because we want a significant *p*-value only if the gene is significant based on both classical *p*-value-based and the more modern effect-size-based meta-analysis. The *p*-values are corrected for multiple comparisons using an FDR approach. Finally, a predefined threshold is used to select the genes that are differentially expressed or methylated.

### 2.2. Multi-Omics Data Integration

This section describes the third and fourth modules of the framework. The inputs of the third module (Figure 1C) are two lists of genes obtained from the meta-analysis step described in section 2.1 above. The first list includes the differentially expressed genes (DEGs), while the second one includes the differentially methylated genes (DMGs) across the given phenotypes. From these two lists of genes, we first select the genes that are present in both lists, i.e., the genes that are both differentially expressed and methylated. Next, we filter them by selecting the genes for which the mRNA and methylation changes occurred in opposite directions. This is motivated by the fact that methylation correlates negatively with gene expression (Shafi et al., 2018). In other words, when a CpG site is methylated in the promoter regions, it typically represses the transcriptional activity of that region by restricting the binding of specific transcription factors (TFs). Alternatively, when a CpG site is unmethylated in the promoter regions, it allows for the binding of those TFs (Jones, 2012). Finally, we identify the methylation-driven genes (MDGs) by filtering the genes that have unsigned effect sizes lower than a given threshold. This is an optional step of the framework. The default threshold is set to zero (no filtering).

Identified MDGs can be thought of as individual gene markers that can distinguish the phenotypes of a given disease, based on both individual mRNA and methylation data. However, to better understand the underlying disease mechanisms, and to better predict patient prognosis, it is important to incorporate known information about the interactions between the genes (Mitra et al., 2013).

The fourth module of the framework (Figure 1D) uses the identified MDGs, DEGs and the given network information to identify the subnetworks that are perturbed by the signals propagated through the edges of the MDGs. For each MDG, we create its own DE neighborhood by selecting the DEGs that are directly connected with it. All identified subnetworks are then merged together into a larger network. This concept of network propagation has been used by several research groups for active subnetwork identification using transcriptomic data (Komurov et al., 2012; Ansari et al., 2017) and mutational hotspot identification in human cancers (Ciriello et al., 2012). Finally, within this larger network, we select the genes that are part of the largest *cliques* as our proposed signature. This idea is driven by the fact that cliques are fully-connected subnetworks in which all nodes are connected in a pairwise fashion; and therefore, genes that are part of a clique are more likely to be functionally related (Pradhan et al., 2012).

### 2.3. Perturbation Clustering

In order to evaluate the prognostic value of the proposed signature, we use the genes present in the signature to identify disease subtypes from the independent patient cohort. For clustering, we use PINS (Nguyen et al., 2017b; Nguyen H. et al., 2018) to perform perturbation clustering that was developed in our research lab for tumor subtyping. PINS can automatically determine the number of clusters and then identify subtypes that are the most stable against noise and data perturbation. PINS is developed based on the observation that small changes in any kind of quantitative assay will be inherently present between individuals, even in a truly homogeneous population in the absence of any molecular subtypes. Therefore, well-defined subtypes of a disease have to be stable with respect to small changes in the measured values. In order to identify robust subtypes, PINS repeatedly perturbs the data by adding Gaussian noise and then clusters the patients. PINS yields subtypes and patient patterns that are least affected by data perturbation. More details of the algorithm can be found in Nguyen et al. (2017b).

Here, the input of the subtyping algorithm is a matrix in which the rows represent the patients and the columns represent the signature genes identified by our framework. Different gene signatures yield different matrices (same set of patients/rows but different sets of genes/columns). We expect that a better signature will provide better subtyping, i.e., subtypes with more significant survival differences. The number of clusters (*k*) is automatically determined by PINS. We simply used the default settings of the PINS R package (Nguyen H. et al., 2018).

## 3. Results

We demonstrate the performance of the proposed framework by constructing network-based signatures for two diseases: glioblastoma multiforme (GBM) and low-grade glioma (LGG). In the GBM study, we included only the stage IV glioma tumors, whereas in the LGG study we included stage II and III glioma tumors. This is consistent with others such as TCGA (Cancer Genome Atlas Research Network et al., 2015), Noushmehr et al. (2010) and Garkavtsev et al. (2004), who also considered stage II and III glioma tumors as LGG. All staging is based on the World Health Organization (WHO) standard. All discovery datasets used in this manuscript were obtained from GEO (Barrett et al., 2005). Dataset summaries and preprocessing techniques are described in the Supplementary Materials. We downloaded the protein-protein interaction (PPI) networks from the STRING database version 10.5 to obtain information about the gene interactions. STRING provides a confidence score (ranging from 0 to 1,000) for each interaction in the network. Here we used a score of 900 to select the high confidence interactions, resulting in a network of 9,941 genes and 227,186 interactions (top 4.9% interactions).

One of the most widely accepted techniques to evaluate the prognostic performance of a gene signature is to test its ability to predict patients' survival in independent datasets (Chang et al., 2005; Shedden et al., 2008; Szász et al., 2016). In order to achieve this goal, we used PINS (described in section 2.3) on independent gene expression validation datasets obtained from three different sources: (i) TCGA, (ii) GEO, and (iii) CGGA (Yan et al., 2012; Sun et al., 2014). None of these datasets have been used in the original training datasets. PINS can automatically determine the number of clusters (denoted by k). We use only the list of genes present in the proposed subnetwork as features, instead of all genes present in the datasets. Survival analysis is performed using Kaplan–Meier survival analysis (Kaplan and Meier, 1958) and their statistical significance is assessed using a Cox regression model (Cox, 1972).

### 3.1. Glioblastoma (GBM) Study

We first identify 2,183 DEGs by performing *leave-one-out* meta-analysis (section 2.1) on four mRNA datasets (GSE7696, GSE4290, GSE90598, and GSE22866). Similarly, we analyze five methylation datasets (GSE60274, GSE22867, GSE50923, GSE79122, and GSE36278) and identify 1,205 DMGs. These nine discovery datasets include a total of 622 samples: 533 samples from GBM patients and 89 from healthy (non-tumor) individuals. Descriptions of these datasets are provided in Table S1. We use a stringent threshold of 0.1% for both differential expression and methylation.

**Figure 2:**
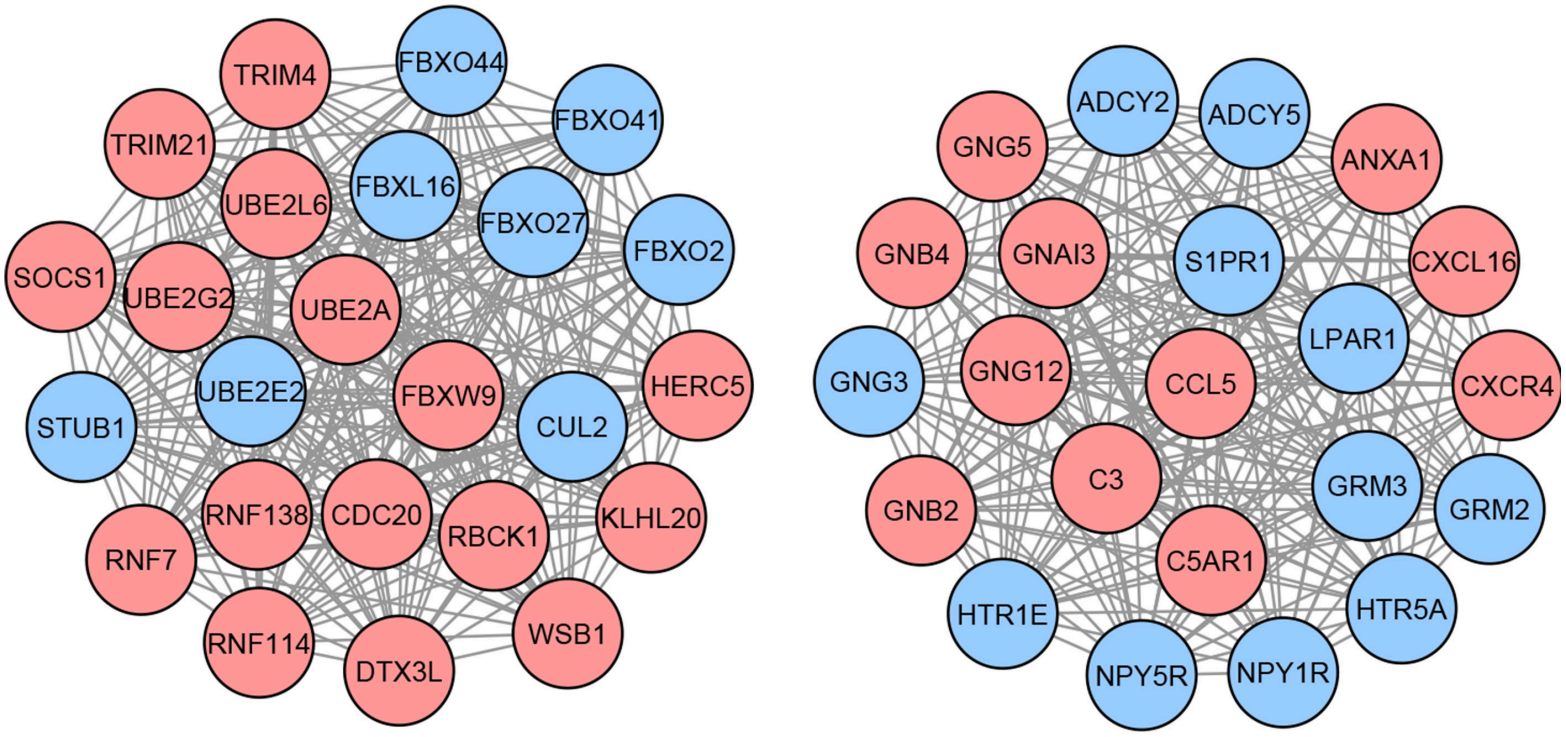
Proposed network-based signature for GBM, containing a total of 46 genes organized in two different cliques. Each node in this graph represents a gene, while each edge describes the interaction between a gene pair. The interactions are retrieved from the STRING database. The colors of the nodes represent the effect sizes obtained from the meta-analysis step described in Figure 1A: red represents genes with a positive effect size while blue represents genes with a negative effect size.

Next, we identify the list of methylation-driven genes (MDGs) based on the three following criteria: (i) genes present in the list of DEGs with absolute mRNA effect sizes >1, (ii) genes present in the list of DMGs with absolute methylation effect sizes >1, and (iii) genes that have opposite mRNA and methylation effect sizes (i.e., genes with positive mRNA effect sizes need to have negative methylation effect sizes, while genes with negative mRNA effect sizes need to have positive methylation effect sizes). The identified list contains 45 MDGs. Each of these identified MDGs are then used as seeds in the network propagation step to build neighbor networks of DEGs (section 2.2). These subnetworks are then merged together to form a larger network, containing a total of 214 candidate genes. Finally, within the larger network, the largest cliques contain 46 genes which constitute the proposed network-based signature for this disease (Figure 2).

We demonstrate the utility of the proposed signature on two independent gene expression datasets; one, downloaded from the TCGA GBM cancer site (The Cancer Genome Atlas Research Network, 2013), contains gene expression profiles of 525 individual patients, and the other one, GSE4412 (Freije et al., 2004), was downloaded from GEO and contains gene expression profiles of 59 individual patients. For both datasets, our proposed signature combined with PINS is able to identify two groups of patients with significantly different survival rates using the Cox regression model. The Cox *p*-value for TCGA datasets is 7.38E-04, whereas the Cox *p*-value for GSE4412 is 9.70E-03.

We compare our signature with the following 7 previously published GBM gene signature panels: 9 methylation-based gene signature proposed by Shukla et al. (2013), 13 methylation-based gene signature proposed by Etcheverry et al. (2010), 14 prognostic gene signature proposed by Arimappamagan et al. (2013), 35 methylation based gene signature proposed by Smith et al. (2014), 35 prognostic gene signature proposed by Fatai and Gamieldien (2018), 36 methylation-based gene signature proposed by Chiang et al. (2014) and 48 gene signature proposed by Crisman et al. (2016).

**Figure 3:**
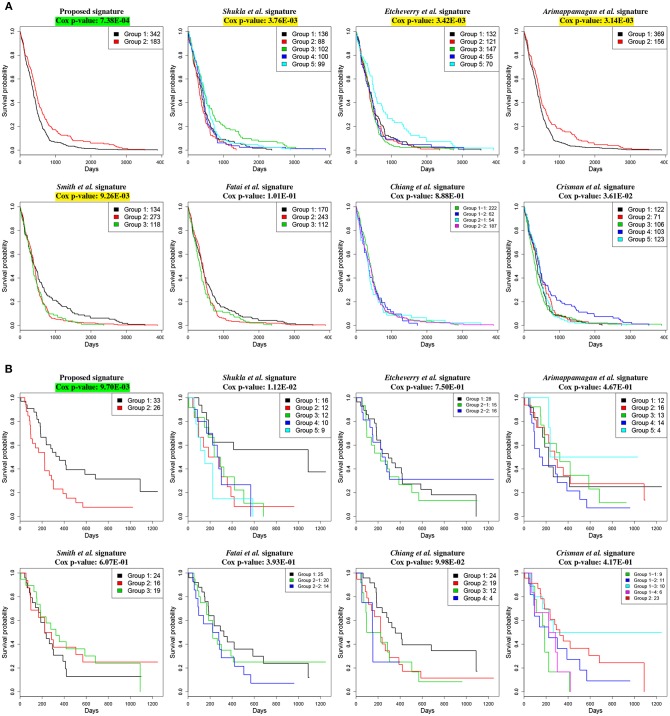
Kaplan–Meier survival analysis on GBM studies, using different gene signature panels. **(A)** TCGA dataset which contains gene expression profiles from 525 individual patients. **(B)** GSE4211 dataset which contains gene expression profiles from 59 individual patients. The horizontal axes represent the time (in days) from the start of the study, whereas the vertical axes represent estimated survival percentage. Yellow colors represent the Cox *p*-values that are significant (< 0.01). The green color indicates the best signature (i.e., lowest Cox *p*-value) for the given dataset. These results show that the proposed signature yields the best separation between aggressive and less aggressive disease on both datasets.

The comparison based on the prognostic performances of these gene signature panels is shown in Table 1. Related survival curves are shown in Figure 3. PINS identifies the optimal number of clusters based on the given input, which is denoted by *k* in the table. The cells highlighted in yellow represent the Cox *p*-values that are significant (< 0.01). The cells highlighted in green show the best signature (i.e., lowest Cox *p*-value) for each dataset. These results show that in both datasets, the proposed signature achieves the best results. Furthermore, in the GSE4412 dataset, only the proposed signature is able to achieve a significant Cox *p*-value.

**Table 1:** Prognostic performance of different gene signature panels related to GBM.

		**TCGA**		**GSE4412**	
		**(525 patients)**		**(59 patients)**	
**Gene signatures**	**Number of genes**	***k***	**Cox *p*-value**	***k***	**Cox *p*-value**
Proposed signature	46	2	7.38E-04	2	9.70E-03
Shukla et al.	9	5	3.76E-03	5	1.12E-02
Etcheverry et al.	13	5	3.42E-03	3	7.50E-01
Arimappamagan et al.	14	2	3.14E-03	5	4.67E-01
Smith et al.	35	3	9.26E-03	3	6.07E-01
Fatai et al.	35	3	1.01E-01	3	3.93E-01
Chiang et al.	36	4	8.88E-01	4	9.98E-02
Crisman et al.	48	5	3.61E-02	5	4.17E-01

*Clustering is performed by using PINS. The number of clusters identified by the algorithm is denoted by k. The cells highlighted in yellow represent the Cox p-values that are significant (< 0.01). The cells highlighted in green represent the best signature (i.e., lowest Cox p-value) for each dataset. These results indicate that the proposed signature is able to achieve the lowest Cox p-values on both independent datasets*.

### 3.2. Low-Grade Glioma (LGG) Study

Similar to the previous study, here we perform *leave-one-out* meta-analysis on five mRNA datasets (GSE16011_cohort1, GSE16011_cohort2, GSE4290, GSE68848, and GSE4271) and three DNA methylation datasets (GSE90496, GSE109379, and GSE53227), and identify 1,564 DEGs and 2,721 DMGs respectively. These eight datasets contain a total of 1,787 samples. Among them, 1,026 samples are from LGG patients while 761 from either GBM patients or healthy (non-tumor) individuals. Descriptions of these datasets are provided in Table S2. In this study, we use a threshold of 5% for differential expression and methylation.

**Figure 4:**
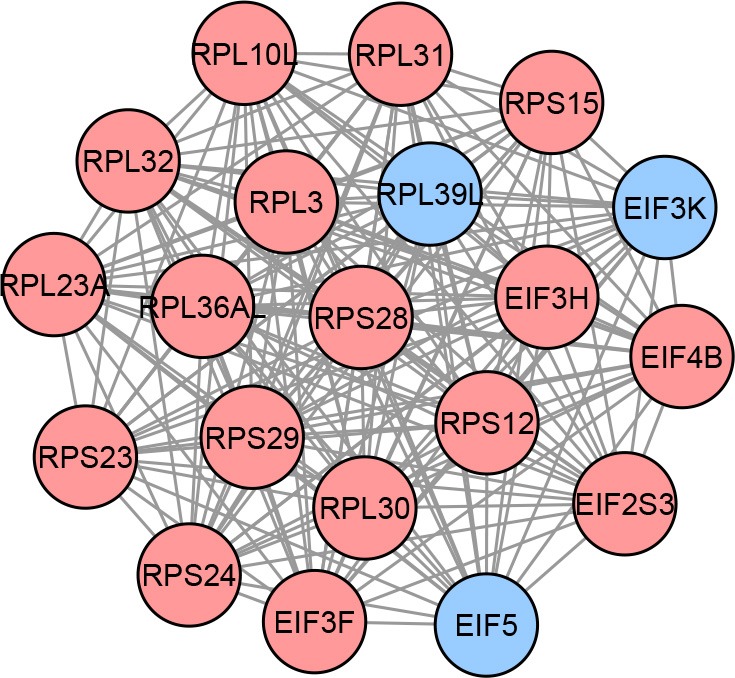
Proposed network-based signature for LGG, containing a total of 20 genes organized in a clique. Each node in this graph represents a gene, while each edge describes the interaction between a gene pair. The interactions are retrieved from the STRING database. The colors of the nodes represent the effect sizes obtained from the meta-analysis step described in Figure 1A: red represents genes with a positive effect size while blue represents genes with a negative effect size.

After integrating DEGs and DMGs in the third module, we find 52 methylation-driven genes (MDGs). Next, we perform network propagation to construct the subnetworks that contain the DEGs directly connecting to MDGs. After merging these subnetworks, we obtain a list of 110 candidate genes. Finally, 20 genes are selected based on the maximum clique present in the network which is the proposed signature for this study. The identified network-based signature is shown in Figure 4.

To demonstrate the utility of the proposed signature, we use two independent gene expression datasets; one from TCGA LGG cancer site (Cancer Genome Atlas Research Network et al., 2015) that contains a total of 515 patients, and the other one from CGGA that contains a total of 170 patients. We use PINS to perform a perturbation clustering using the genes present in the proposed network as features. Similar to the GBM study, for both datasets, the groups of patients identified based on the given signature have significantly different survival profiles. For the TCGA dataset, the Cox *p*-value is 5.48E-09 with 4 clusters whereas for the CGGA dataset, the Cox *p*-value is 1.82E-04 with 5 clusters.

**Figure 5:**
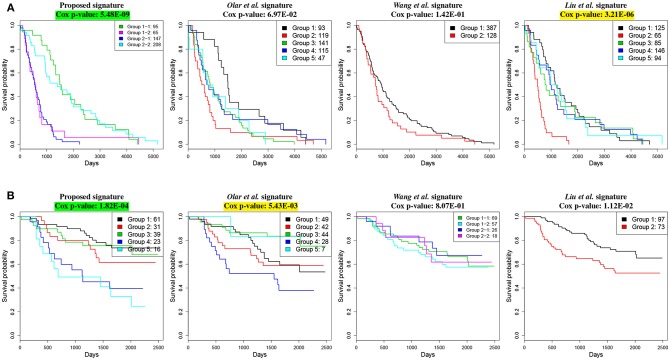
Kaplan–Meier survival analysis on LGG studies, using different gene signature panels. **(A)** TCGA dataset which contains gene expression profiles from 515 individual patients. **(B)** CGGA dataset which contains gene expression profiles from 170 individual patients. The horizontal axes represent the time (in days) from the start of the study, whereas the vertical axes represent estimated survival percentage. Yellow colors represent the Cox p-values that are significant (< 0.01). The green color indicates the best signature (i.e., lowest Cox p-value) for the given dataset. These results show that the proposed signature yields the best separation between aggressive and less aggressive disease on both datasets.

We compare our proposed signature with the following 3 published LGG gene signature panels: a set of 6 genes identified by Olar and Sulman (2015), a meta-signature of 20 genes proposed by Wang et al. (2017) and a panel of 24 genes proposed by Liu et al. (2011). The comparison between the results obtained with these signatures is shown in Table 2. The related survival curves are shown in Figure 5. In the TCGA dataset, the proposed signature and the signature proposed by Liu et al. achieve significant Cox p-values. In CGGA dataset, significant Cox p-values are achieved by the proposed signature and the signature proposed by Olar et al. These results show that in both datasets, the proposed signature achieves the best results.

**Table 2:** Prognostic performance of different gene signature panels related to LGG.

		**TCGA**		**CGGA**	
		**(515 patients)**		**(170 patients)**	
**Gene signatures**	**Numer of genes**	***k***	**Cox *p*-value**	***k***	**Cox *p*-value**
Proposed signature	20	4	5.48E-09	5	1.82E-04
Olar et al.	6	5	6.97E-02	5	5.43E-03
Wang et al.	20	2	1.42E-01	4	8.07E-01
Liu et al.	18	5	3.21E-06	2	1.12E-02

Clustering is performed by using PINS. The number of clusters identified by the algorithm is denoted by k. The cells highlighted in yellow represent the Cox p-values that are significant (< 0.01). The cells highlighted in green represent the best signature (i.e., lowest Cox p-value) for each dataset. These results indicate that the proposed signature is able to achieve the lowest Cox p-values on both independent datasets.

### 3.3. Network-Based Signature vs. Methylation-Driven Genes (MDGs)

To demonstrate the contribution of the network information in our framework, we compare the prognostic performance of the proposed network-based signature with the performance of a signature derived from methylation-driven genes (MDGs) alone. Table 3 shows the Cox *p*-values obtained by using these two types of signatures on the four independent datasets used in the above two studies. PINS was used to group the samples. For GBM, the MDGs and the proposed signature contain 45 and 46 genes respectively, while for LGG, the MDGs and the proposed signature contain 27 and 20 genes, respectively. Results indicate that, for both diseases (each disease contains two independent datasets), network-based signatures outperform the individual markers (i.e., MDGs) based on their ability to predict the patients' clinical outcome.

**Table 3:** Prognostic performance of network-based signatures vs. individual markers.

	**GBM study**					**LGG study**				
		**TCGA GBM**		**GSE4412**			**TCGA LGG**		**CGGA**	
**Gene signatures**	**m**	**k**	**Cox P**	**k**	**Cox P**	**m**	**k**	**Cox P**	**k**	**Cox P**
Meth. driven genes (MDGs)	45	4	9.36E-03	3	1.18E-01	27	3	3.22E-06	2	1.43E-03
Network-based signature	46	2	7.38E-04	2	9.70E-03	20	4	5.48E-09	5	1.82E-04

*Clustering is performed by using PINS. Number of clusters identified for a given dataset is denoted by k, while the number of genes for a given study is denoted by m. Cells highlighted in green represent the best signature (i.e., lowest Cox p-value) for each dataset. Results indicate that incorporating network information leads to better prognostic gene markers*.

## 4. Discussion

One widely used technique to combine multiple independent studies is to perform a *horizontal* meta-analysis (i.e., combining sample-unmatched studies of the same data type). This approach is unable to combine studies coming from multiple data types. Hence, it is not suitable for the identification of the *mechanism of action* of a given disease. Another technique is to perform a *vertical* meta-analysis (i.e., combining sample-matched studies from multiple data type) which accounts for the heterogeneity that may arise across different *omic* layers. However, the latter technique requires each data type to be available for each individual patient, which is expensive and impractical for the studies with large sample sizes. To overcome these challenges, in this manuscript, we propose a multi-cohort and multi-omics meta-analysis framework that identifies network-based signatures using independent mRNA and DNA methylation studies available in the public repositories. The identified signatures are evaluated based on their ability to distinguish patients with different survival profiles on independent validation datasets.

One of the inputs required for the proposed framework is the known interactions between the genes. This information can come from any protein-protein interaction database for the given organism and is independent of the specific experiment or condition. In our case, this type of data came from the STRING database, which would be suitable for any experiment involving more than 2,000 organisms. The discovery datasets used in this manuscript are downloaded from GEO. We have included all gene expression and methylation studies related to GBM and LGG that have a total number of samples measuring 20 or more after data preprocessing. Datasets from any other resources such as TCGA, ArrayExpress (Rustici et al., 2013), etc., can also be used as long as they contain samples from two phenotypes (disease vs. normal, treated vs. non-treated, etc.). The framework is appropriate for the disease conditions whose mechanisms of actions are known to be triggered by the change in DNA methylation. Due to the important role of DNA methylation in glioma (Heyn and Esteller, 2012; Turcan et al., 2012), we demonstrate our proposed framework on two subtypes of glioma; the most aggressive one, GBM, and the comparatively less aggressive LGG. However, this framework can be used to identify network-based markers for other disease conditions as well.

We leverage the concept of the network propagation algorithms mentioned in Mitra et al. (2013) to identify candidate subnetworks from the methylation-driven genes. The final network-based markers are selected based on the maximum clique. Cliques are complete graphs in which all nodes are connected in a pairwise fashion, and therefore, genes that are part of a clique are likely to be functionally related. In previous years, the utility of using cliques has been demonstrated in multiple disease conditions such as breast cancer (Shi et al., 2010), colorectal cancer (Pradhan et al., 2012), etc. Other subnetwork identification techniques, such as greedy algorithms (e.g., PinnacleZ, Chuang et al., 2007), clustering-based methods (e.g., SAMBA, Tanay et al., 2004), scoring based on centrality measurements (e.g., Wang et al., 2011), etc., can be utilized as well. A comprehensive review of the currently available tools for subnetwork identification can be found in Nguyen et al. (2019).

We investigate how the groups of patients identified in the TCGA GBM dataset, using our proposed signature (Figure 3A), relate with the available histopathological variables or treatments. Table S3 shows the confusion matrix of the two groups of patients associated with the proposed GBM signature and the five GBM subtypes recognized by the original authors (The Cancer Genome Atlas Research Network, 2013). Enrichment analysis using Fisher's Exact Test (FET) indicates that the group of patients with lower survival rate is enriched with Mesenchymal subtype (*p* = 1.04E-19), whereas the group of patients with higher survival rate is associated with Proneural (*p* = 1.98E-14) subtype and G-CIMP tumors (*p* = 4.27E-10). This confirms the fact that G-CIMP tumors belong to the Proneural subtype (Noushmehr et al., 2010; Verhaak et al., 2010). In addition, the better survival group is enriched with IDH1 mutation (*p* = 1.80E-06) and relatively younger patients (Wilcoxon rank sum (WRS) test *p* = 0.01), which is also acknowledged by others (Noushmehr et al., 2010; The Cancer Genome Atlas Research Network, 2013). Furthermore, we investigate patients' responses to Temozolomide (TMZ), a drug which is FDA approved for the treatment of GBM. We do this by calculating the survival Cox *p*-value for each group (the better survival group and the lower survival group) based on the patients treated with and without TMZ (treated with other drugs or untreated). The results indicate that only one group of patients (not both) is associated with favorable TMZ drug response, which is reflected by significantly different survival rates of the drug-responders and the drug-resistants (Cox *p*-value = 7.34E-06). Our finding explains why it has previously been noted that there is a group of patients who do not respond well to TMZ (Kitange et al., 2009; Lee, 2016).

Similarly, to investigate the groups of patients identified on TCGA LGG, we obtained clinical information from TCGA that includes three subtypes of glioma: IDH wild-type, IDH mutant-codel, and IDH-mutant-non-codel (Ceccarelli et al., 2016). Enrichment analysis using FET reveals that the groups of patients with lower survival rates (cluster “1-2” and “2-1” in Figure 5A) are enriched with wild-type IDH (*p* = 2.30E-16 and 1.94E-06) and MGMT promoter unmethylation (*p* = 4.99E-06 and 0.001). These results confirm the findings previously reported by TCGA and others (Hegi et al., 2005). In addition, we found that the lower survival rates are associated with a higher tumor purity score (WRS *p*-value = 0.007). Previously, it has been shown by others that a higher tumor purity score is associated with tumor growth, disease progression and drug resistance (Yoshihara et al., 2013).

**Figure 6:**
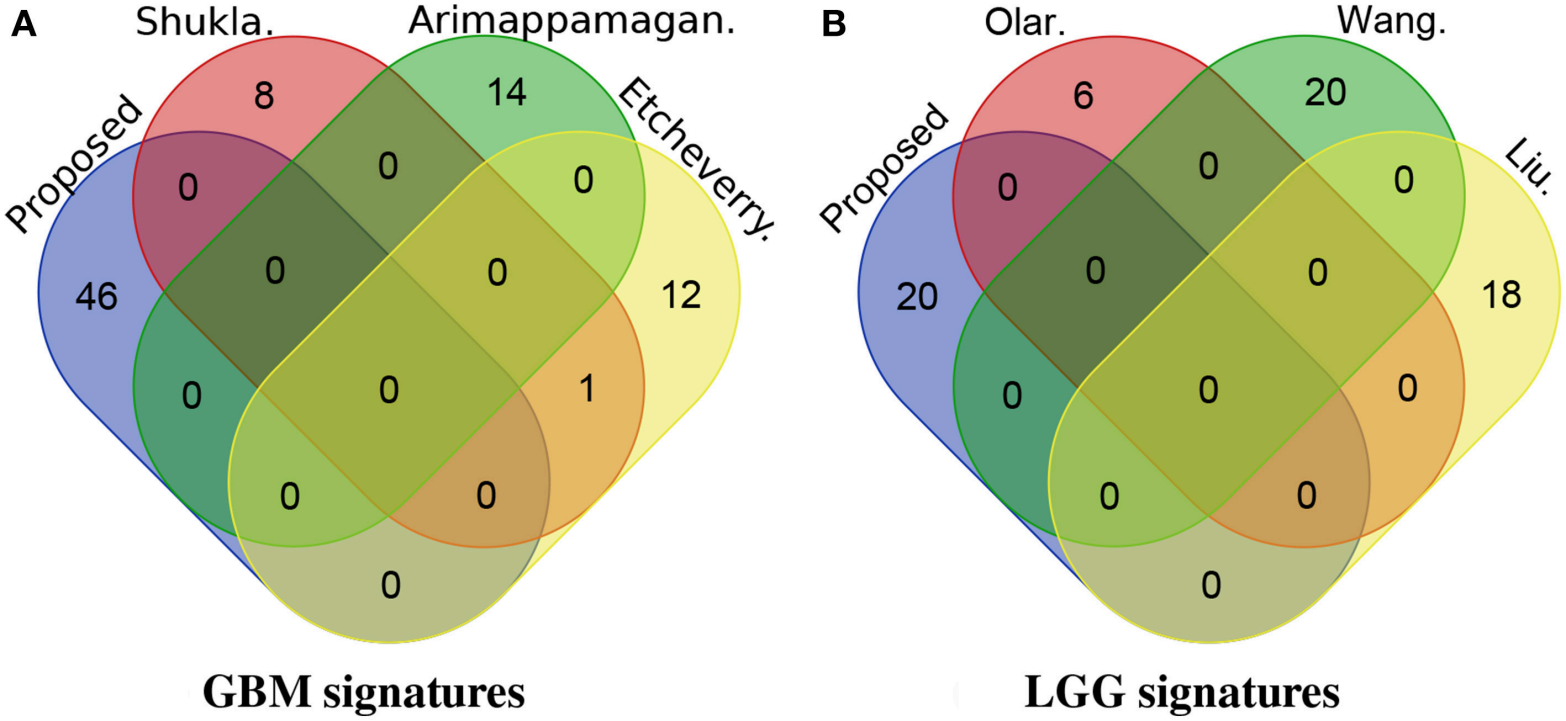
The overlap between the proposed signatures and other previously published signatures. For GBM, none of the genes proposed in this manuscript are present in the signatures proposed by Shukla et al., Etcheverry et al., and Arimappamagan et al. Similarly for LGG, none of the genes proposed in this manuscript are present in the signatures proposed by Olar et al., Wang et al., and Liu et al. **(A)** GBM signatures. **(B)** LGG signatures.

We also investigate the novelty of our identified signatures by checking their overlap with other published signature genes (Figure 6). For GBM, none of the genes proposed in this manuscript are present in the other three top (based on the Cox *p*-value on TCGA dataset) gene signature panels (i.e., panels of gene signatures proposed by Shukla et al., Etcheverry et al., and Arimappamagan et al.). Similarly for LGG, none of the genes proposed in this manuscript are present in the panels of gene signatures proposed by Olar et al., Wang et al., and Liu et al.

One of the main reasons for this is that the types of evidence used by our proposed framework are different from other relevant studies. Our proposed framework identifies gene signatures using evidence from three different sources: (i) mRNA expression, (ii) DNA methylation, and (iii) protein-protein interactions (PPI). In addition, it combines heterogeneous independent studies within each data type (mRNA and DNA methylation) using an effect-size-based meta-analysis approach. In contrast, none of the relevant studies identify their gene signatures considering all three types of evidence that we used. They are based on frameworks that either do not integrate information from multiple data levels or do not combine multiple studies within one data level, or both. Therefore, a very small or no overlap between the signatures proposed by our framework and the signatures proposed by other relevant studies is to be expected. Furthermore, the existing signatures have little or no overlap among themselves, even though many of them are based on the same type of evidence. In spite of the fact that our proposed genes have not been previously reported, they provide the best ability to distinguish between aggressive and less aggressive disease in all independent datasets that we used.

Importantly, our proposed GBM signature contains several genes that play crucial roles in the underlying mechanisms of GBM. For instance, according to Deng et al. (2016), ADCY2 is known to be involved in the progression of diffuse intrinsic pontine glioma; ANXA1 has shown to be involved in GBM apoptosis by Festa et al. (2013); Pan et al. (2017) demonstrated that CCL5 is responsible for creating an autocrine circuit for Mesenchymal GBM growth; Xie et al. (2015) investigated the role of CSC20 and found its crucial role in tumor-initiating cell (TIC) proliferation in GBM; CXCR4, LPAR1 and TRIM21 play important roles GBM cell proliferation as demonstrated by Ehtesham et al. (2009), Loskutov et al. (2018), and Lee et al. (2017), respectively; Kim et al. (2018) demonstrated the therapeutic role of RNF138 in GBM; Mahajan-Thakur et al. (2017) reviewed the role of S1PR1 in GBM and found that its over-expression is associated with improved GBM prognosis; SOCS1 plays a vital role as a tumor suppressor in GBM, as investigated by Baker et al. (2009); STUB1 has shown to be involved in glioma cell proliferation by Syed et al. (2015); etc. Similarly, our proposed LGG signature contains genes that are known to be related to glioma. For instance, according to Shi et al. (2006), EIF3F is downregulated in most human tumors including glioma; EIF5 and RPS12 are known to be involved in brain metastasis in primary breast tumors (Sanz-Pamplona et al., 2011); Shahbazian et al. (2010) has shown that EIF4B is a potential target for anti-cancer therapies; etc.

Furthermore, we use iPathwayGuide (Advaita Corporation, 2019) to perform an extensive pathway analysis to identify the mechanisms captured by the proposed signatures. iPathwayGuide uses an impact analysis that calculates the true impact of a pathway by combining two types of evidence. The first type of evidence is the classical over-representation of DE genes in each pathway. The second type of evidence captures several other important biological factors such as the position of all the genes on each pathway, the magnitude of their expression change, the direction and type of the signals transmitted between genes as described by the pathway, etc. The impact analysis has been shown to be able to identify the significantly impacted pathways much better than classical over-representation alone (Drăghici et al., 2007; Tarca et al., 2009).

**Figure 7:**
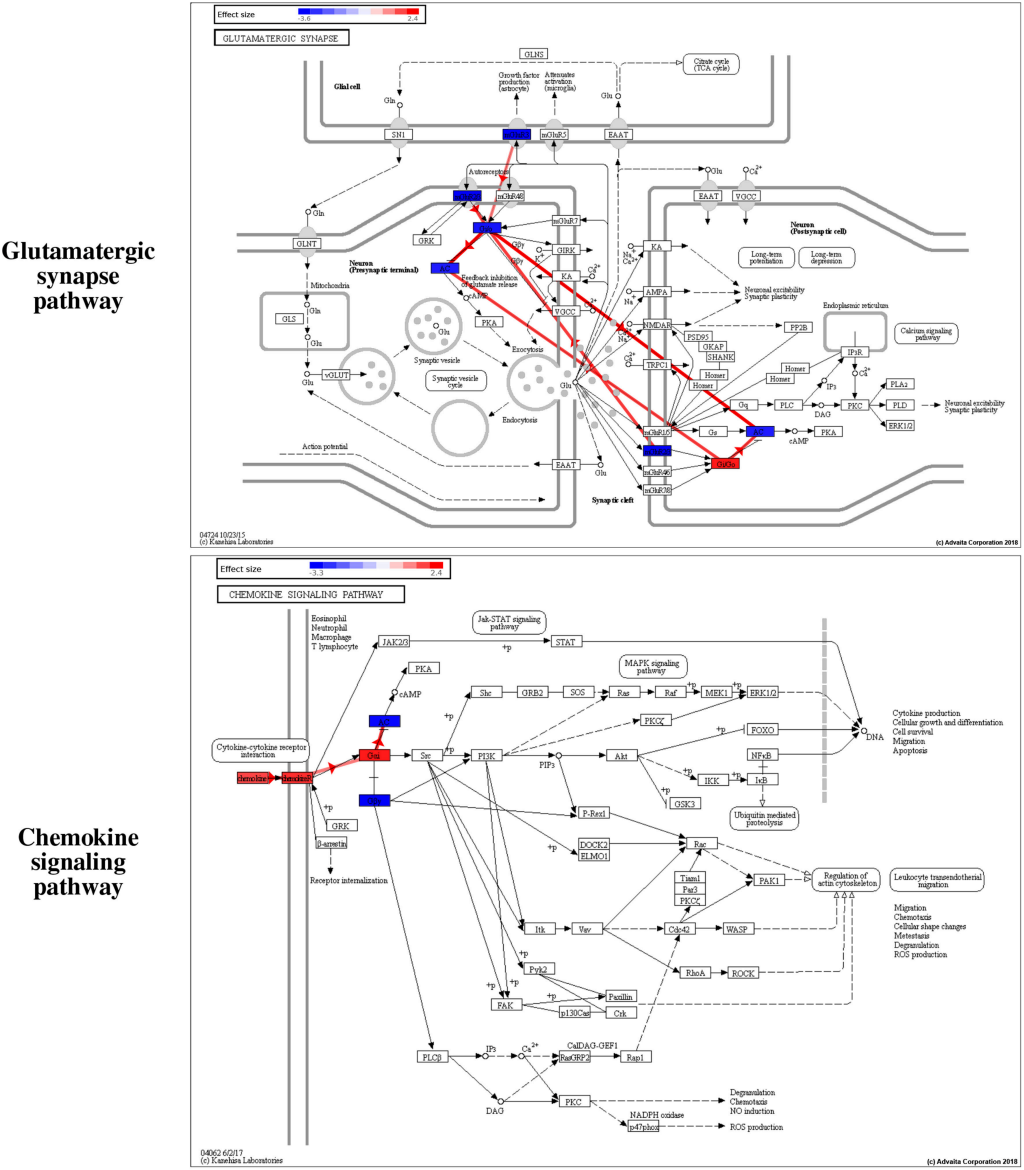
Interesting putative mechanisms are identified by iPathwayGuide (www.advaitabio.com) on the **Glutamatergic synapse** and the **Chemokine signaling** pathways. The colors of the nodes represent the effect sizes obtained from the meta-analysis step described in Figure 1A of the manuscript: red represents genes with a positive effect size while blue represents genes with a negative effect size. The edges highlighted in red represent the *coherent edges* between the genes, which indicate the edges for which the measured effect changes are consistent with the phenomena described by the pathway.

**Figure 8:**
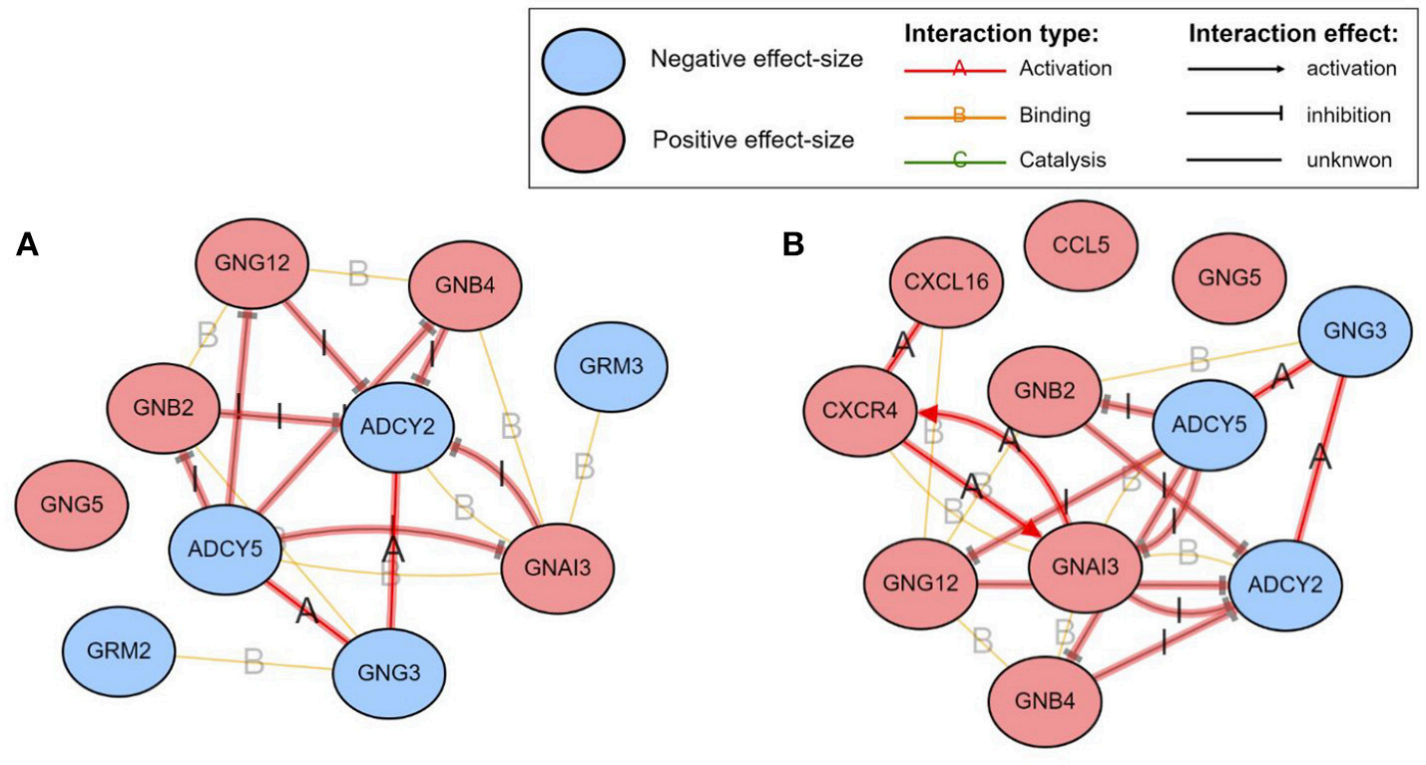
The mechanisms involving the proposed **GBM signature** in the **Glutamatergic synapse (A)** and the **Chemokine signaling (B)** pathways, as identified by iPathwayGuide (www.advaitabio.com). The colors of the nodes represent the effect sizes obtained from the meta-analysis step described in Figure 1A. The edges represent molecular actions between the genes obtained from the STRING database. The edges highlighted in red indicate the *coherent edges* between the genes.

Among the pathways reported as significant, interesting putative mechanisms are identified by the impact analysis on the Glutamatergic synapse pathway and the Chemokine signaling pathway. These are shown in Figure 7. The colors of the nodes represent the effect sizes obtained from the meta-analysis step described in Figure 1A: red represents genes with a positive effect size while blue represents genes with a negative effect size. The edges highlighted in red represent *coherent edges*. A coherent edge is an edge for which the measured effect changes are consistent with the phenomena described by the pathway. For example, if gene A inhibits gene B, and if gene A is upregulated, gene B is expected to be downregulated. If the measured changes are consistent with this inhibition, the edge corresponding to this interaction is referred to as being coherent. Several such coherent edges form coherent chains of perturbation propagation which can be thought of as putative mechanisms. Figure 8 shows a closer look of the coherent edges within the two pathways mentioned above.

For LGG, two pathways are significantly impacted with the proposed gene signature after correcting for multiple comparisons: the Ribosome pathway and the RNA transport pathway (Figures S1, S2). The reason for having only two pathways as significantly impacted could be explained by the fact that LGG is an early stage of glioma and, therefore, the differences across the given phenotypes are not reflected in the pathway level.

## 5. Conclusion

In an effort to identify disease-specific biomarkers that can explain the underlying biological mechanism and predict associated patients' survival, several computational approaches have been proposed over the past few years. The majority of the approaches have limited clinical applicability since they do not fully utilize the crucial information that is currently available in public repositories. In this manuscript, we propose an integrative framework that is able to identify network-based biomarkers for a given disease condition, utilizing information from three different sources: (i) multiple independent mRNA studies, (ii) multiple independent DNA methylation studies and (iii) protein-protein interactions. We demonstrate the utility of the proposed framework by constructing subnetworks related to GBM and LGG, using 17 independent mRNA and DNA methylation studies containing a total of 2,409 samples. We validate our proposed signatures on four independent gene expression datasets containing a total of 1,269 patients. The results indicate that our proposed network-based signatures are able to better predict patients' survival than other published signatures for these diseases.

## Author Contributions

AS and SD conceived of and designed the project. AS implemented the method in R and performed the data analysis and all computational experiments. TN, AP, and HN helped AS to perform the data analysis. AS and SD wrote the manuscript. All authors reviewed the manuscript.

### Conflict of Interest Statement

The authors declare that the research was conducted in the absence of any commercial or financial relationships that could be construed as a potential conflict of interest.
