# Supplementary material for: A Multi-Cohort and Multi-Omics Meta-Analysis Framework to Identify Network-Based Gene Signatures
Source: Front Genet. 2019 Mar 19;10:159. doi: 10.3389/fgene.2019.00159 (PMC6434849; doi:10.3389/fgene.2019.00159)
Supplement: Supplementary file 1 [file Data_Sheet_1.pdf]

frontiers

Supplementary Material: A multi-cohort and multi-omics meta-analysis framework to identify network-based gene signatures

1 DATASET DESCRIPTION

All the discovery datasets are downloaded from Gene Expression Omnibus (GEO) (Barrett et al., 2005), while all the independent validation datasets are obtained from GEO, TCGA [http://cancergenome.nih.gov] and CGGA (Sun et al., 2014; Yan et al., 2012). Summary of the datasets for both glioblastoma (GBM) and low-grade glioma (LGG) are provided in the Table S1 and S2.

Table S1. Summary of the datasets used for GBM study. 9 independent datasets containing a total of 622 samples (533 GBM vs 89 normal) were used as discovery datasets whereas 2 independent datasets containing a total of 584 samples were used for validation purposes.

	Datasets	Discovery/ validation	Data type	Number of samples	Contrast	Platform
1	GSE7696	Discovery	GE	64	59 GBM vs 5 normal	Affymetrix HG U133 Plus 2.0
2	GSE4290	Discovery	GE	100	77 GBM vs 23 normal	Affymetrix HG U133 Plus 2.0
3	GSE90598	Discovery	GE	23	16 GBM vs 7 normal	Affymetrix HG 2.1 ST Array
4	GSE22866	Discovery	GE	46	40 GBM vs 6 normal	Agilent-WHG Microarray 4x44K
5	GSE60274	Discovery	DM	64	59 GBM vs 5 normal	Illumina HumanMethylation450
6	GSE22867	Discovery	DM	60	56 GBM vs 4 normal	Illumina HumanMethylation27
7	GSE50923	Discovery	DM	78	54 GBM vs 24 normal	Illumina HumanMethylation27
8	GSE79122	Discovery	DM	45	36 GBM vs 9 normal	Illumina HumanMethylation450
9	GSE36278	Discovery	DM	142	136 GBM vs 6 normal	Illumina HumanMethylation450
10	TCGA (GBM)	Validation	GE	525	525 GBM	Illumina HiSeq/GASeq RNASeq
11	GSE4412	Validation	GE	59	59 GBM	Affymetrix HG U133 A

Table S2. Summary of the datasets used for LGG study. 8 independent datasets containing a total of 1,787 samples (1,026 LGG vs 761 others) were used as discovery datasets whereas 2 independent datasets containing a total of 642 samples were used for validation purposes.

	Datasets	Discovery/ validation	Data type	Number of samples	Contrast	Platform
1	GSE16011_C1	Discovery	GE	117	109 LGG vs 8 normal	Affymetrix HG U133 Plus 2.0
2	GSE16011_C2	Discovery	GE	268	109 LGG vs 159 GBM	Affymetrix HG U133 Plus 2.0
3	GSE4290	Discovery	GE	99	76 LGG vs 23 normal	Affymetrix HG U133 Plus 2.0
4	GSE68848	Discovery	GE	243	215 LGG vs 28 normal	Affymetrix HG U133 Plus 2.0
5	GSE4271	Discovery	GE	100	24 LGG vs 76 GBM	Affymetrix HG U133 A
6	GSE90496	Discovery	DM	420	301 LGG vs 119 normal	Illumina HumanMethylation450
7	GSE109379	Discovery	DM	428	104 LGG vs 324 GBM	Illumina HumanMethylation450
8	GSE53227	Discovery	DM	112	88 LGG vs 24 GBM	Illumina HumanMethylation27
9	TCGA (LGG)	Validation	GE	515	515 LGG	Illumina HiSeq/GASeq RNASeq
10	CGGA	Validation	GE	170	170 LGG	Agilent-WHG Microarray 4x44K

2 DATA PREPROCESSING AND NORMALIZATION

The R programming language is used to generate the results included in this manuscript.

2.1 Gene expression datasets

For all the gene expression datasets from GEO, we download the raw probe level data and apply the same normalization procedure to make it consistent. Eight out of nine datasets are from Affymetrix platform, which are normalized using RMA background adjustment, quantile normalization and median polish summarization. We use the *threestep* function from *affyPLM* package to achieve this goal Bolstad (2004). For probe to gene mapping, standard genome wide annotation packages are used from bioconductor. Median values are taken whenever multiple probes mapped to the same gene. One dataset is from Agilent platform, which is normalized using *limma* package.

For the TCGA validation datasets, we download preprocessed mRNASeq data. We removed the samples that have more than 10% missing genes. We then removed the genes that have missing values in any of the remaining samples. For the CGGA validation dataset, we download the normalized dataset coming from Agilent-WHG Microarray 4x44K platform. Median values are taken whenever multiple probes mapped to the same gene.

2.2 DNA methylation datasets

For three out of eight DNA methylation datasets (GSE60274, GSE90496, and GSE109379), raw IDAT files were available. All three of these datasets are coming from Illumina Infinium HumanMethylation 450K platform. We normalize these datasets using the *preprocessFunnorm* function from *minfi* package (Aryee et al., 2014). For the other five DNA methylation datasets are coming from both Illumina Infinium Hu-manMethylation 27K and 450K platforms. For these datasets, we download the normalized probe level beta values.

After normalization, methylation levels of the CpG sites are quantified using beta values which is ranged from 0 to 1. A value close to 0 denotes low methylation level whereas a value close to 1 denotes high methylation level. CpG sites with missing values were removed from further analysis. For 27K datasets, we removed the CpG sites that are on the sex chromosomes and map probes to genes. For probe to gene mapping, standard genome wide annotation packages are used from bioconductor. Median values are taken whenever multiple probes mapped to the same gene.

For 450K datasets, we remove the CpG sites: (i) that are located on the sex chromosomes, (ii) that contain known SNPs, and (iii) that have lower detection p-value in more than 10% of the samples. While estimating methylation levels of the genes, we require each gene to have at least 3 CpG situes while each of these CpG sites to fall in transcription start sites (TSS) or $5'$ untranslated region ($5'$ UTR) or 1st exon. Since the platform contains multiple CpG sites mapping to one gene, we collapsed the CpG sites that map to a single gene by taking their median methylation value. This procedure can be replaced with any other sophisticated function such as taking the CpG sites that are correlated each other.

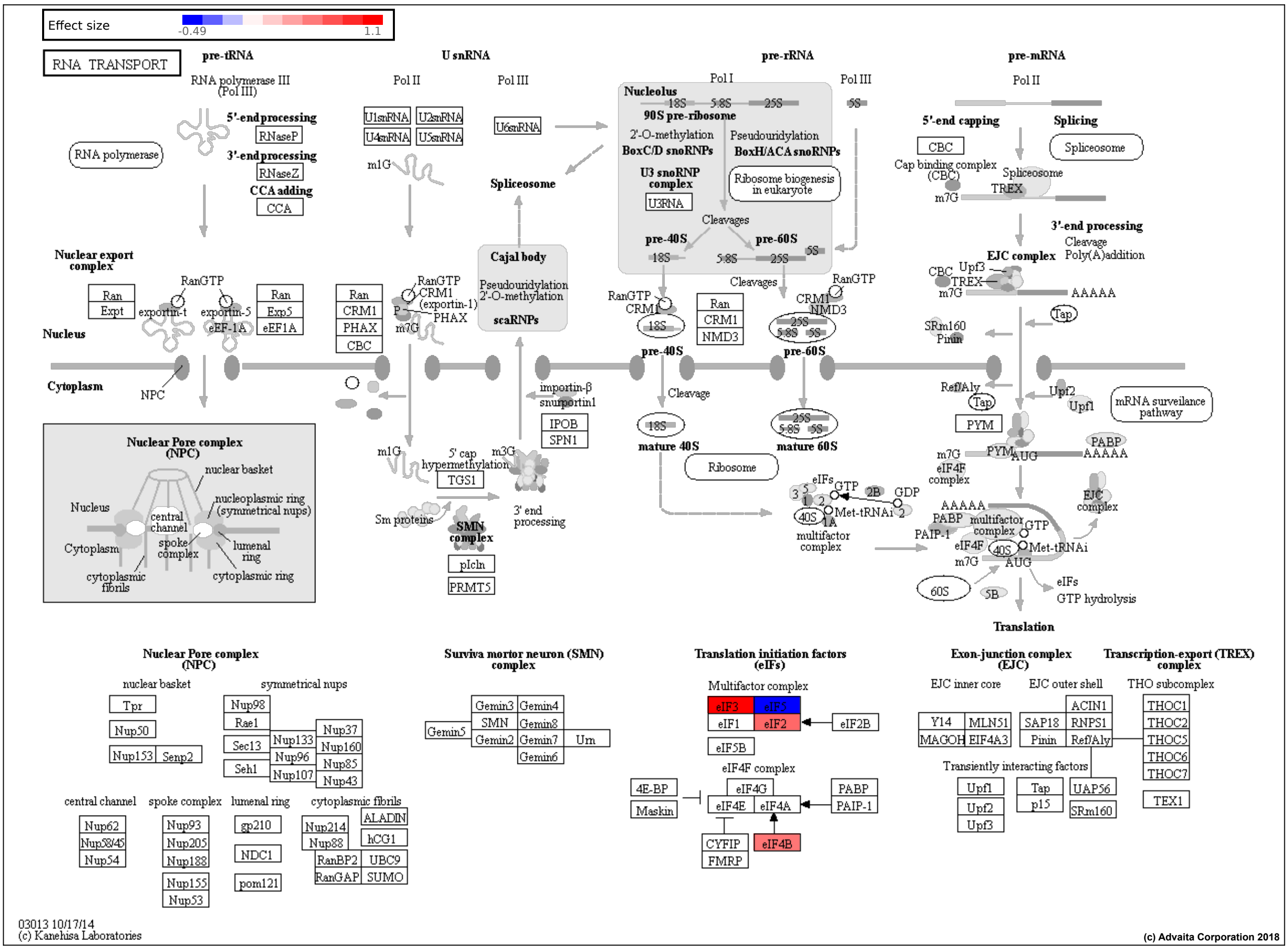

Figure S1. The **RNA transport pathway** is significantly impacted with the proposed gene signature related to **LGG**. The colors of the nodes represent the effect sizes obtained from the meta-analysis step described in Figure 1A of the manuscript: red represents genes with a positive effect size while blue represents genes with a negative effect size.

Table S3. Confusion matrix of the two groups of patients identified by PINS clustering using the proposed network-based GBM signature and the five GBM subtypes identified by TCGA (The Cancer Genome Atlas Research Network, 2013)

	Cluster 1	Cluster 2
Classical	101	43
G-CIMP	7	32
Mesenchymal	143	12
Neural	59	24
Proneural	31	68

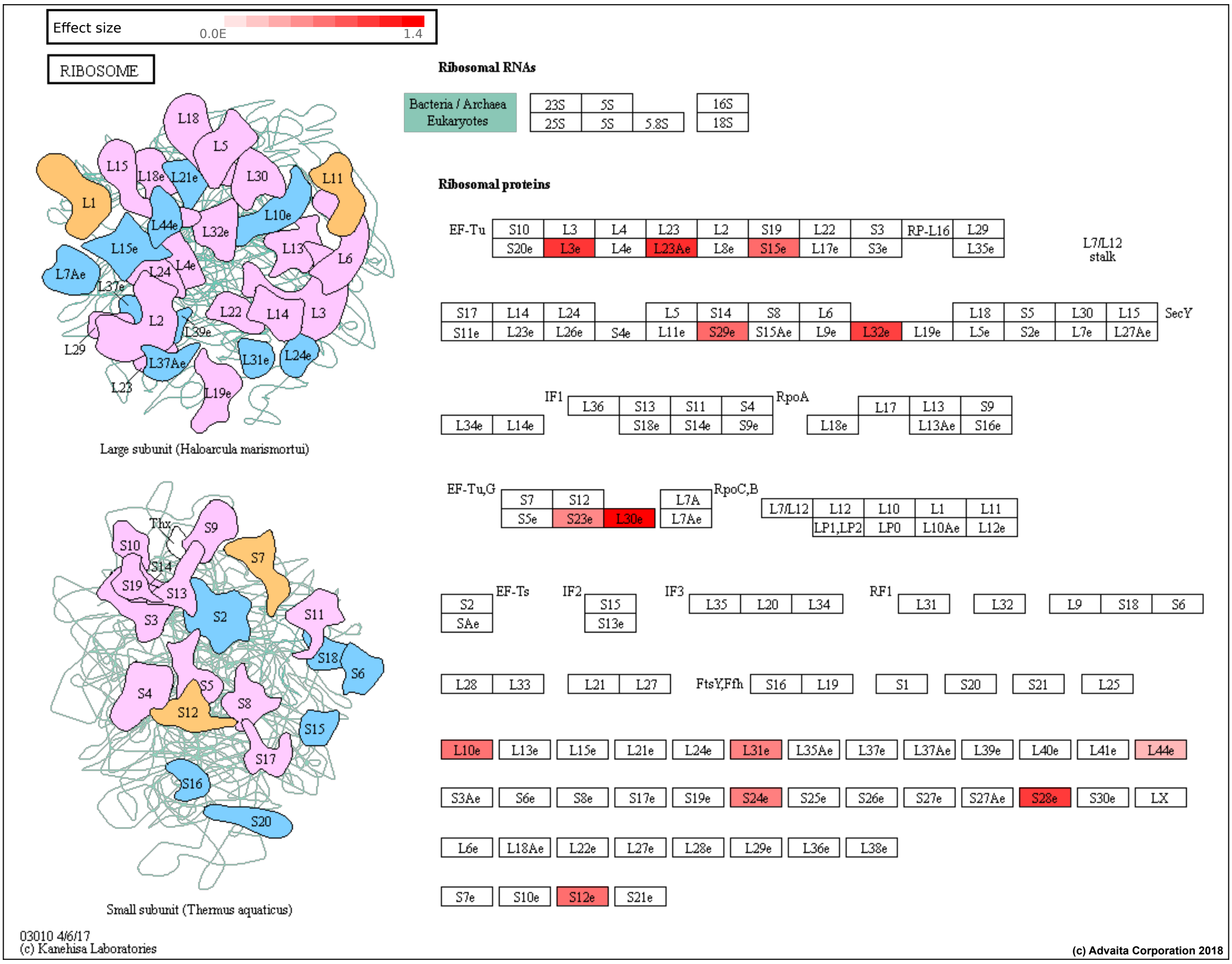

Figure S2. The **Ribosome pathway** is significantly impacted with the proposed gene signature related to **LGG**. The colors of the nodes represent the effect sizes obtained from the meta-analysis step described in Figure 1A of the manuscript: red represents genes with a positive effect size while blue represents genes with a negative effect size.

Table S4. The list of 46 genes present in the proposed network-based signature for GBM

ADCY2	DTX3L	GNG12	NPY1R	TRIM4
ADCY5	FBXL16	GNG3	NPY5R	UBE2A
ANXA1	FBXO2	GNG5	RBCK1	UBE2E2
C3	FBXO27	GRM2	RNF114	UBE2G2
C5AR1	FBXO41	GRM3	RNF138	UBE2L6
CCL5	FBXO44	HERC5	RNF7	WSB1
CDC20	FBXW9	HTR1E	S1PR1	
CUL2	GNAI3	HTR5A	SOCS1	
CXCL16	GNB2	KLHL20	STUB1	
CXCR4	GNB4	LPAR1	TRIM21	

Table S5. The list of 20 genes present in the proposed network-based signature for LGG

EIF2S3	RPL31
EIF3F	RPL32
EIF3H	RPL36AL
EIF3K	RPL39L
EIF4B	RPS12
EIF5	RPS15
RPL10L	RPS23
RPL23A	RPS24
RPL3	RPS28
RPL30	RPS29

REFERENCES

Aryee, M. J., Jaffe, A. E., Corrada-Bravo, H., Ladd-Acosta, C., Feinberg, A. P., Hansen, K. D., et al. (2014). Minfi: a flexible and comprehensive Bioconductor package for the analysis of Infinium DNA methylation microarrays. *Bioinformatics* 30, 1363–1369

Barrett, T., Suzek, T. O., Troup, D. B., Wilhite, S. E., Ngau, W. C., Ledoux, P., et al. (2005). NCBI GEO: mining millions of expression profiles–database and tools. *Nucleic Acids Research* 33, D562–6

Bolstad, B. M. (2004). *Low-level analysis of high-density oligonucleotide array data: background, normalization and summarization*. Ph.D. thesis, University of California

Sun, Y., Zhang, W., Chen, D., Lv, Y., Zheng, J., Lilljebjörn, H., et al. (2014). A glioma classification scheme based on coexpression modules of EGFR and PDGFRA. *Proceedings of the National Academy of Sciences* 111, 3538–3543

The Cancer Genome Atlas Research Network (2013). The somatic genomic landscape of glioblastoma. *Cell* 155, 462–477. doi:10.1016/j.cell.2013.09.034

Yan, W., Zhang, W., You, G., Zhang, J., Han, L., Bao, Z., et al. (2012). Molecular classification of gliomas based on whole genome gene expression: a systematic report of 225 samples from the Chinese Glioma Cooperative Group. *Neuro-oncology* 14, 1432–1440
